# *Toxoplasma gondii:* CD8 T Cells Cry for CD4 Help

**DOI:** 10.3389/fcimb.2019.00136

**Published:** 2019-05-01

**Authors:** Imtiaz A. Khan, SuJin Hwang, Magali Moretto

**Affiliations:** Department Microbiology, Immunology and Tropical Medicine, The George Washington University, Washington, DC, United States

**Keywords:** toxoplasma, CD8T cells, CD4T cells, IL-12, IL-21, BLIMP-1, exhaustion

## Abstract

*Toxoplasma gondii*, an apicomplexan parasite, is a pathogenic protozoan that can infect the central nervous system. In pregnant women, infection can result in congenital problems of the fetus, while in immunocompromised individual it can lead to severe neurological consequences. Although CD8 T cells play an important effector role in controlling the chronic infection, their maintenance is dependent on the critical help provided by CD4 T cells. In a recent study, we demonstrated that reactivation of the infection in chronically infected host is a consequence of CD8 T dysfunction caused by CD4 T cell exhaustion. Furthermore, treatment of chronically infected host with antigen-specific non-exhausted CD4 T cells can restore CD8 T cell functionality and prevent reactivation of the latent infection. The exhaustion status of CD4 T cells is mediated by the increased expression of the transcription factor BLIMP-1, and deletion of this molecule led to the restoration of CD4 T cell function, reversal of CD8 exhaustion and prevention of reactivation of the latent infection. In a recent study from our laboratory, we also observed an increased expression of miR146a levels by CD4 T cells from the chronically infected animals. Recent reports have demonstrated that microRNAs (especially miR146a) has a strong impact on the immune system *of T*. *gondii* infected host. Whether these molecules have any role in the BLIMP-1 up-regulation and dysfunctionality of these cells needs to be investigated.

## Introduction

*Toxoplasma gondii*, an Apicomplexan with broad geographical representation, can cause severe infection of the central nervous system (Torgerson et al., 2014). Also, maternal infection during pregnancy can result in congenital infection with serious neurological and ocular complications (Peyron et al., 2011). In immune compromised individuals, reactivation of latent neurologic foci can result in encephalitis (TE) (Saadatnia and Golkar, 2012). *T. gondii* is an important foodborne pathogen found in many domesticated animals used for human consumption in United States (Hill and Dubey, 2013; Dubey et al., 2014) and human infection often develops after the ingestion or handling of undercooked or raw meat containing tissue cysts (Hoffmann et al., 2012). Alternatively, it can result from direct contact with cat, soiled cat litter or from the consumption of water or food contaminated by oocysts excreted in the faces of infected cats (Torrey and Stanley, 2013). Toxoplasmosis continues to be a significant public health problem worldwide and while more than a million people are believed to be infected with this parasite in the United States, about 3,000 individuals develop symptomatic disease annually. The cost of illness caused by *T. gondii* in the United States of America is estimated to be nearly 3 billion with an annual loss of 11,000 quality adjusted life years (QALY) worldwide (Batz et al., 2012). Recent studies have also linked *T. gondii* infection to mental illness like schizophrenia and suicidal tendencies in apparently asymptomatic individuals (Pedersen et al., 2012; Torrey et al., 2012; Bhadra et al., 2013a,b). Reactivation of latent toxoplasmosis can have severe consequences not only in individuals infected with human immunodeficiency virus, but also in patients who have undergone allogeneic hematopoietic stem cell transplant (HSCT) or received solid organ transplant (Tenter et al., 2000; Bhopale, 2003). Although toxoplasmosis presents most often as a localized central nervous system infection, severely immunocompromised patients like those receiving HSCT also exhibit disseminated infection involving lungs, heart, and liver (Tenter et al., 2000). In recent studies, it was reported that severe disseminated toxoplasmosis in patients undergoing HSCT and leading to Intensive care Unit admission had a poor prognosis, necessitating strategies aimed at preventing this fatal opportunistic infection (Schmidt et al., 2013; Voegele et al., 2013). As far as HIV infected population is concerned, despite combination antiretroviral therapy (cART) many patients continue to suffer from toxoplasmosis. Furthermore, even after full cART introduction, 65% of these patients died within the first year of diagnosis with TE (Mayor et al., 2011). Overall, *T. gondii* infection induces a strong immune response in the infected host that restricts the infection to latency (Jordan and Hunter, 2010). However, in case this immunity is compromised it can pose a severe risk to infected individuals and lead to reactivation of infection (Shearer et al., 1986).

## Protective Immune Responses Against *T. Gondii* Infection

Innate immune responses that include NK cells, neutrophils and dendritic cells are important for the resistance against the parasite (Yarovinsky, 2014). Original work revealed that antibodies transferred from infected to naïve hamsters provided little protection to the recipient (Frenkel, 1967). Also, in this study, intact or lysed spleen cells were transferred from infected to naïve animals which were subsequently challenged. It was observed that only intact cells could confer protective immunity to the recipient animals, emphasizing the role of cell mediated immunity in this response. A later study using a vaccine strain model of infection demonstrated that both CD4 and CD8 T cells are important for controlling the infection, even though CD8 played a more dominant role (Suzuki and Remington, 1988). Further studies, using an antibody depletion method, demonstrated a pivotal role for IFNγ, a major mediator of protective immunity against the disease (Suzuki et al., 1988). Shortly thereafter, Khan and colleagues developed for the first time, antigen-specific CD8 T cell clones capable of responding and killing *T. gondii* tachyzoites via cytotoxic activity *in vitro* (Suzuki et al., 1988; Khan et al., 1994). These reports suggest that two effector mechanisms, including IFNγ mediated activation of macrophages and cytotoxicity mediated by CD8 T cells, play a role in controlling *T. gondii* infection. Studies conducted years later suggest that while IFNγ plays a critical role in controlling *T. gondii* infection during the acute phase of infection (Suzuki et al., 1988; Gazzinelli et al., 1992), chronicity of the infection is contained by cytotoxic CD8 T cells (Suzuki et al., 2010). Subsequently, Suzuki's group reported that antigen-specific CD8 T cells are capable of removing cysts from immunodeficient animals infected with *T. gondii* (Sa et al., 2017). Moreover, the importance of CD8 T cells in the protection against *T. gondii* infection was also demonstrated by other laboratories. In one of these reports, CD8 CTL (cytotoxic T lymphocytes) generated against a vaccine strain of the parasite protected the animals against a lethal challenge with a virulent strain of the parasite (Gazzinelli et al., 1991). In another study conducted by Brown et al., the importance of CD8 T cells in controlling toxoplasma cyst burden was demonstrated (Brown et al., 1994). A number of other studies have confirmed the dominant role of CD8 T cells in long-term immunity against *T. gondii* infection, which is important to keep the chronic infection under control (Gazzinelli et al., 1992; Khan et al., 1994; Khan and Kasper, 1996). The mechanism of CD8 T cell mediated protection during the late stages of the acute infection can be attributed to their ability to produce IFNγ (Gazzinelli et al., 1992), which plays a pivotal role in immune-protection against *T. gondii* infection (Suzuki et al., 1988). However, during chronic toxoplasmosis, the perforin dependent cytotoxic ability of CD8 T cell population is involved in restricting the parasite to chronic state (Suzuki et al., 2010).

### CD8 T Cell Immunity During *T. gondii* Infection

Effector CD8 T cells are one of the important sources of IFNγ, which is responsible for controlling both the acute as well as the chronic phase of the infection (Suzuki and Remington, 1988; Gazzinelli et al., 1992; Khan et al., 1999). The importance of CD8 T cells in maintaining chronic toxoplasmosis was demonstrated by antibody depletion studies. In these experiments, treatment of chronically infected mice with anti-CD8 antibody led to the reactivation of the latent infection (Gazzinelli et al., 1992; Bhadra et al., 2011a). However, the biggest challenge in restricting the infection to the chronic phase is the fact that CD8 T cells need to be maintained in a functional state for a sustained period of time. Studies conducted in an TE model have demonstrated that during the later stages of the chronic infection, CD8 T cells exhibit a graded increase in the expression of inhibitory receptor PD-1 resulting in their dysfunctionality/exhaustion (Bhadra et al., 2011a). CD8 T-cell exhaustion has been reported in several chronic viral infections, like LCMV infection, and are characterized by persisting high levels of viremia (Mueller and Ahmed, 2009; Shin et al., 2009). Similar to these viral infections, blockade of the PD-1–PDL-1 pathway in mice carrying chronic toxoplasma infection reinvigorates the suboptimal CD8 T-cell response, resulting in the control of parasites reactivation and prevention of mortality (Bhadra et al., 2012). Interestingly, in continuation of these studies, it was observed that PD-1 is preferentially expressed by polyfunctional memory CD8 T cells, which leads to their loss of functionality and renders them susceptible to apoptosis (Bhadra et al., 2012). The selective dysfunctionality in the memory CD8 population could be an impediment for the development of a robust CD8 T cell response needed for the long-term protection against the infection. Another question that needs to be addressed is if apoptosis of the CD8 T cell population is dependent on the strain of parasites as previously reported (Nishikawa et al., 2007; Hippe et al., 2009). In that case, the role of different parasite strains in the induction of CD8 T cell dysfunctionality will need to be ascertained. However, as stated in our previous manuscript (Bhadra et al., 2011b) a major hurdle in investigating these questions thoroughly was the lack of information regarding *T. gondii* dominant CD8 epitopes. Nevertheless, the decapeptide HPGSVNEFDF (HF10) from the dense granule protein GRA6 as a naturally processed peptide recognized by CD8 T cells during *T. gondii* infection in BALB/c (H-2d) mice has been identified (Blanchard et al., 2008). This was subsequently followed by the discovery of two more H2-Ld-restricted epitopes, SPMNGGYYM and IPAAAGRFF, from the dense granule protein GRA4 and rhoptry protein ROP7 (Frickel et al., 2008). Subsequently, Wilson et al. identified a novel H-2Kb-restricted epitope, SVLAFRRL, derived from TGD057, a protein of unknown function (Wilson et al., 2010). However, as we have stated earlier (Bhadra et al., 2011b) with the discovery of as MHC class I tetramers, T-cell receptor transgenic mice and ovalbumin expressing transgenic parasites will enable the investigation of the effector mechanisms from various CD8 subsets and their correlation with immune protection during acute and chronic phases of the infection with much greater clarity.

### Role of CD4 T Cells in the Induction of CD8 T Cell Response

CD4 T cells are critical for the induction of primary CD8 T cell response (Bennett et al., 1997). CD8 T cell immunity generated in the absence of CD4 T cells cannot be maintained and respond poorly to secondary challenge (Bennett et al., 1997; Laidlaw et al., 2016). CD4 T cells help CD8 T cell response primarily by facilitating antigen-presentation and up-regulation of co-stimulatory molecules on the dendritic cells to optimal levels that induce a robust CD8 T cell response (Bennett et al., 1997; Schoenberger et al., 1998). In addition to their role in primary CD8 T cell immunity, CD4 T cells are also essential for the robust expansion of memory CD8 T cell population (Williams et al., 2006a). Helpless CD8 T cells upon re-stimulation undergo activation induced cell death and memory response is severely impeded (Janssen et al., 2005). CD4 T cells are a critical source of IL-2 which is important for CD8 T cell development (Williams et al., 2006b). Regulatory CD4 T cells (Treg) have been reported to modulate IL-2 exposure of effector CD8 T cells during the primary phase and are essential for generation of functional memory CD8 population (McNally et al., 2011). Similarly role of T follicular helper cells (Tfh) population, which are the primary source of IL-21 (Hale and Ahmed, 2015) in the maintenance of CD8 T cell functionality is well documented (Yi et al., 2009).

## Helper role of CD4 T cell in the maintenance of CD8 functionality

*Toxoplasma gondii* induces a strong CD4 T cell response that is a major source of IFNγ during both acute, as well as chronic infection (Gazzinelli et al., 1992; Liesenfeld et al., 1996), and is similar to what has been observed in other infections (Green et al., 2013). Earlier studies, including those from our laboratory, have demonstrated the importance of CD4 T cells for the maintenance of the CD8 T cell response against intracellular pathogens (Carvalho et al., 2002; Casciotti et al., 2002; Williams et al., 2006a). It is believed that CD8 T cells play an important effector role during *T. gondii* infection, while the CD4 T cell subset provides the essential help needed for their maintenance. It has been reported that depletion of both CD4 and CD8 T cell populations results in the reactivation of latent toxoplasmosis and, as a consequence, the susceptible animals succumb to TE (Casciotti et al., 2002). Similarly, the emergence of severe toxoplasmosis in patients infected with HIV is concomitant with a decline in CD4 T cell numbers (Shearer et al., 1986). Although depleted CD4 numbers in HIV patients lead to increased susceptibility to TE, most cases of toxoplasmosis in HIV patients occur during the late stage of HIV infection (advanced AIDS), when a deficiency in CD8 T cells is also evident (Shearer et al., 1986). Overall, the depleted CD4 population during the late stages of HIV infection compromises the CD8 T cell immunity against the chronic (latent) toxoplasmosis leading to reactivation of the infection.

As stated above, several studies conducted in our laboratory have demonstrated a severe CD8 T cell dysfunctionality during chronic *T. gondii* infection (Bhadra et al., 2011c, 2012; Gigley et al., 2012). In recent publications, we reported that the dysfunction observed in the CD8 T cell population from chronically infected mice is due to inadequate help caused by CD4 exhaustion (Hwang et al., 2016; Moretto et al., 2017). This was established by our findings which showed that transfer of non-exhausted antigen-specific CD4 T cells to chronically infected mice reversed the CD8 dysfunctionality and prevented the reactivation of the latent infection (Hwang et al., 2016). Comparably, a recent report emphasized that a durable CD4 T cell response is more efficient in promoting a robust CD8 T cell immunity against tumors than targeting the CD8 T cells directly (Melssen and Slingluff, 2017). It is believed that the “strong cytokine producing help” from CD4 T cells facilitates the maintenance of CD8 functionality (Bhadra et al., 2011a) (Phan-Lai et al., 2016). Moreover, understanding the mechanism(s) by which CD4 T cells ensures the maintenance of a robust CD8 T cell immunity will provide important insights into the development of new therapeutic strategies that will allow the maintenance of functional CD8 T cell memory, therefore preventing TE in immunosuppressed patients with latent *T. gondii* infection.

### BLIMP-1 Mediated CD4 T Cell Exhaustion During Chronic Toxoplasmosis

Exhausted CD4 T cell population expressed elevated levels of multiple inhibitory receptors concomitant with reduced functionality and up-regulation of BLIMP-1, a transcription factor. Although BLIMP-1 has been intrinsically linked with CD8 T cell exhaustion (Shin et al., 2009), studies from our laboratory have demonstrated that ablation of this transcription factor in CD4 T cells reverses their exhaustion status and allow them to provide essential help to CD8 T cells. This help from recovered CD4 T cells restores the long-term functionality of the CD8 T cells and prevents the reactivation of the latent infection (Hwang et al., 2016). One of the important questions that need attention is why CD4 T cells during chronic toxoplasmosis become exhausted/dysfunctional? Also, the mechanism of BLIMP-1 mediated CD4 T cell exhaustion that leads to CD8 T cell dysfunctionality needs to be investigated.

### Role of microRNA in Immune Response to *T. gondii* Infection

**Figure 1:**
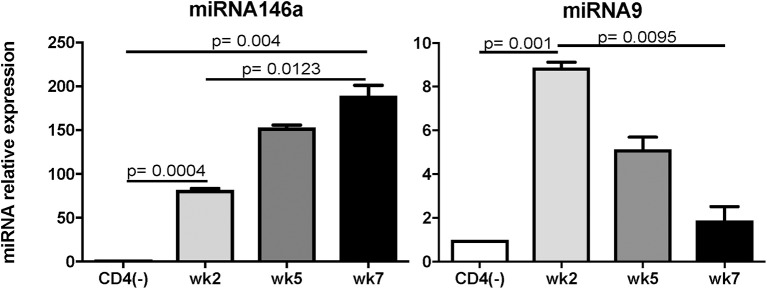
Expression of miRNA146a, and 9 during *T. gondii* infection. C57BL/6 mice were infected per-orally with 10 cysts of Me49 strain of *T. gondii*. Sorted antigen specific CD4 T cells were assayed for miRNA by RT-PCR. The experiment was performed twice. CD4(-) T cells from naïve mice served as controls. Statistical analysis was performed using student's *T*-test.

Recently, studies have demonstrated a pivotal role for microRNAs (miRNAs) in controlling the differentiation as well as functionality of various immune cells (Escobar et al., 2014; Lin et al., 2014). miRNAs are crucial post-transcriptional regulators of hematopoietic cell fate decisions (Oliveto et al., 2017). miR146a and miR 125 have been reported to control the inflammatory response and the outcome of pathogenic infections (Lee et al., 2016). miRNAs were also reported to be the regulators of the host response to infection by apicomplexan parasites (Cai and Shen, 2017). Brain miRNAs changed in abundance in response to *T. gondii* infection (Hu et al., 2018). It has been reported that two immuno-modulatory miRNAs, miR146a and miR155, were co-induced in the brains of mice challenged with *T. gondii* in a strain specific manner (Cannella et al., 2014). We performed a real time PCR on the antigen specific CD4 T cells and found that (a) among the miRNAs tested, miRNA146a was significantly up regulated at week 7 post-infection (p.i.) (Figure 1) a time point at which increased BLIMP-1 expression peaks in these cells (Hwang et al., 2016). Conversely, levels of miRNA9, which has been shown to enhance IL-2 production (Thiele et al., 2012), were increased at week 2 p.i. but were reduced to background levels at week 7 p.i. Based on this information, the role of miR146a in CD4 T cell exhaustion/dysfunctionality clearly needs to be investigated further. As TRAF-6 has been reported to be the direct target of miR146a (Stickel et al., 2014), we suspect that the down-regulation of this molecule by antigen-specific CD4 T cells during chronic toxoplasmosis may lead to increased BLIMP-1 expression. However, before drawing any conclusions, predicted targets of miR146a (www.targetscan.org), including but not limited to TRAF-6, IRAK-1, CD28, Rel, and TLR3 should be investigated even though our data suggests an important role for miR146a in BLIMP-1 mediated CD4 exhaustion. Whether this role is targeted via TRAF-6 or other molecules still need to be determined.

## Role of Co-stimulatory Molecules in Maintaining CD4 T Cell Functionality

It has been postulated that the interplay between positive signals from co-stimulatory molecules and negative inhibitory receptors play an important role in T cell activation, differentiation, effector function, and exhaustion (Chen and Flies, 2013). In this regard, it is important to report that, as stated in a recent review, a robust therapeutic response against infectious diseases or cancer requires not only releasing the brakes (blocking the inhibitory receptors), but also stepping on the gas (targeting the appropriate T cell co-stimulatory molecules) to promote the expansion and functionality of T cells (Linch et al., 2015). Recently, a very important report demonstrated that rescue of exhausted CD8 T cells by PD-1 blockade is exclusively dependent on the interaction between a single co-stimulatory molecule CD28 and its receptor B7 (Hui et al., 2017; Kamphorst et al., 2017). In these studies, it was reported that anti-PD-1 treatment during chronic LCMV infection was ineffective if CD28: B7 interaction was blocked. Thus, the efficacy of inhibitory receptor blockade in reversing T cell exhaustion depends on the up-regulation of important co-stimulatory molecules. However, the involvement of co-stimulatory receptors in the restoration of CD4 functionality due to BLIMP1 ablation has not been studied either in infectious diseases or cancer. These are very important studies and data obtained from these experiments will enable the target of co-stimulatory molecules needed to reverse CD4 T cell dysfunctionality which, in case of Toxoplasmosis and other chronic infections, is important for the maintenance of CD8 T cell immune response. To identify co-stimulatory molecules involved in the rescue of CD4 T cells in the context of BLIMP-1 ablation, we examined a panel of 5 such receptors in CD4 T cells from chronically infected BLIMP-1 conditional knockout mice and wild type littermate controls. 4-1BB, a member of the TNF-TNFR superfamily, was identified as one of the most important co-stimulatory molecules showing high levels of differential expression as compared to wild type littermates (Hwang et al., 2016). A small difference in the expression of another TNFα receptor family member, OX40, was also noted in BLIMP-1 ablated mice while expression of other co-stimulatory molecules (CD27, ICOS, and CD40 L) did not show any significant difference (Hwang and Khan, manuscript in preparation). Treatment of chronically infected animals with 4-1BB agonist antibody prevented host mortality, highlighting the critical importance of this co-stimulatory pathway in the reversal of CD4 T cells exhaustion (Khan et al., 2019). Significantly, treatment of infected animals with a 4-1BB agonist increased the ability of these animals to control parasite multiplication and also increased the ability of infected animals to release IL-21 (Khan et al., 2019), a cytokine produced predominantly by CD4 T cells (Suto et al., 2008) and known to be important for the maintenance of CD8 T cell functionality (Shin et al., 2009). These findings suggest the importance of co-stimulatory molecules in the maintenance of CD4 T cell functionality during a chronic infection, which can be compromised as result of increased BLIMP-1 expression. Obviously, the molecular mechanism(s) involved in BLIMP-1/4-1BB mediated CD4 T cell dysfunction during chronic toxoplasmosis that leads to CD8 exhaustion in chronically infection needs to be studied further.

## Conclusions

**Figure 2:**
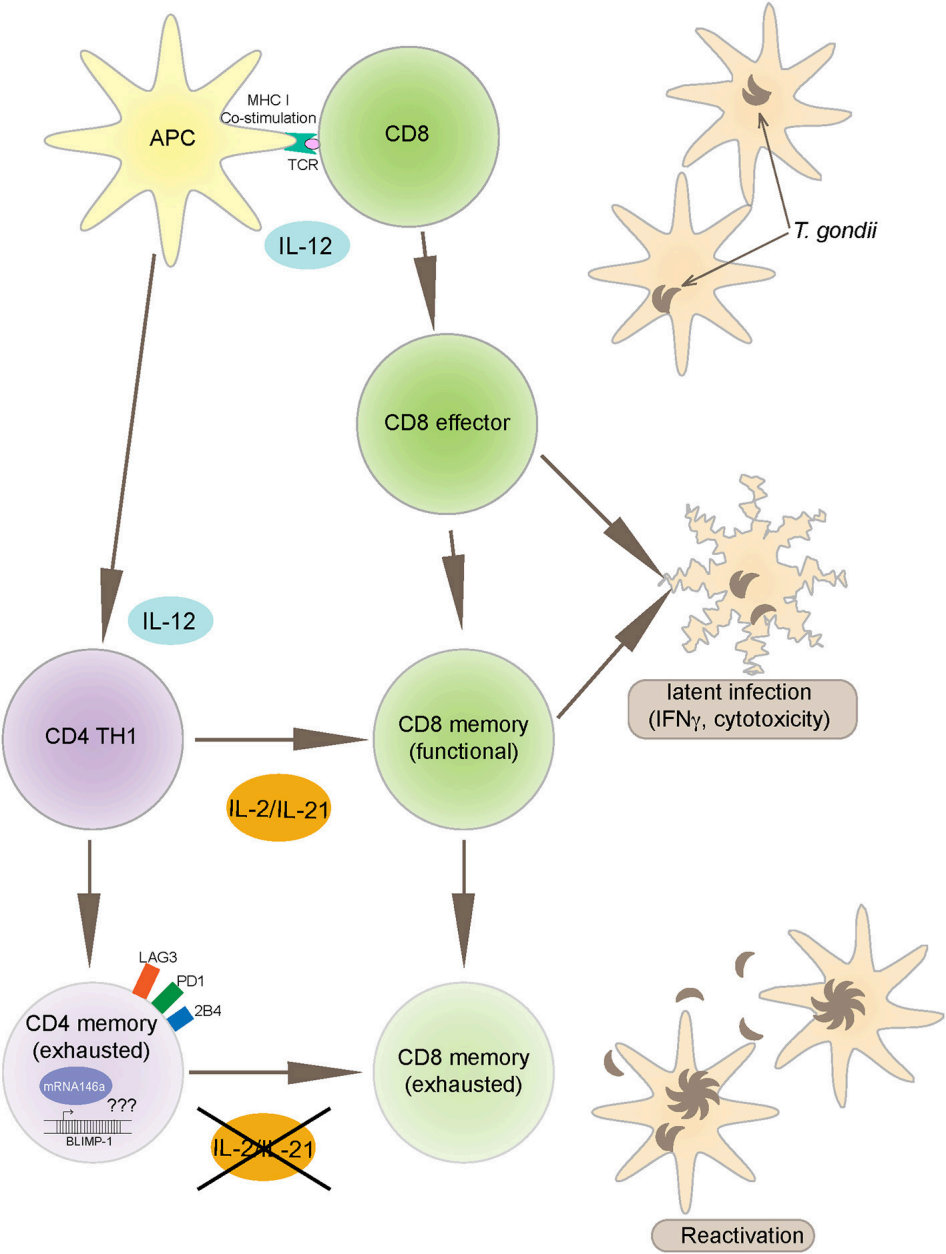
*Toxoplasma gondii* infection induces a strong IL-12 production by APCs (antigen presenting cells) that leads to polarized CD4 (TH1) and robust CD8 T cell effector immunity. CD8 T cells are an important source of IFNγ and also exhibit cytotoxic activity against infected targets, both important mechanisms for controlling the infection. The maintenance of long-term CD8 T cell immunity (memory response) is dependent on CD4 T cell help, mediated primarily by IL-2 and IL-21 production. However, during chronic infection, antigen-specific CD4 T cells express increased BLIMP-1 levels, a transcription factor that leads to up regulation of inhibitory receptors like PD-1. As a consequence to these events, CD4 T cells get exhausted and vital help needed for CD8 T cells is deprived leading to reactivation of infection. The mechanism of BLIMP-1 mediated CD4 exhaustion needs to be investigated. Recent studies from our laboratory reported increased expression of miR146a in the CD4 T cells from chronically infected animals concomitantly to an increase in BLIMP-1. The link between BLIMP-1 and miR146a may provide important insights into the CD4 T cell exhaustion.

*Toxoplasma gondii* infection induces a strong innate and adaptive immune response. While the innate immunity is important for controlling the early stages of the infection (Yarovinsky, 2014), the adaptive immunity is critical for restricting the parasite replication during the later stages (Gazzinelli et al., 1992). Amongst the adaptive immune subsets, CD8 T cells are the primary effector cells while CD4 T cells play an essential helper role to maintain long-term immunity (Casciotti et al., 2002). Notwithstanding, a robust CD8 T cell immunity induced during acute phase of infection, does not result in the total eradication of parasites and the pathogen persists in a chronic state (Bhadra et al., 2011c). Studies conducted in our laboratory have shown that during chronic toxoplasmosis CD8 T cells exhibit increased expression of inhibitory receptors, especially PD-1 that leads to their dysfunction/exhaustion (Bhadra et al., 2011c). In more recent studies, we have demonstrated that CD8 T cell dysfunction is a sequelae of CD4 T cell dysfunction mediated by increased expression of the transcription factor BLIMP-1 (Hwang et al., 2016) In very recent and studies from our laboratory, we observed an increased expression of microRNA146a by antigen-specific CD4 T cells from *T. gondii* (Figure 1), infected animals at the time point at which elevated BLIMP-1 expression in these cells is noted. It will be very interesting and important to determine if there is a BLIMP-1/miR146a axis responsible for CD4 exhaustion during chronic toxoplasmosis. With the use of conditional knock outs for this microRNA it will be essential to determine if the strategies to block miR146a could have a profound effect on BLIMP-1 mediated CD4 T cell functionality that should ensure the persistence of functional CD8 T cell immunity resulting in substantial decreased in chronic parasitic burden (Figure 2). The approach could prove highly beneficial for individuals carrying the infection and chances of reactivation would significantly decrease especially in immunocompromised subjects.

## Ethics Statement

This study was carried out in accordance with the recommendations of the George Washington University Institutional Animal Care and Use Committee under Animal Use Protocol A052.

## Author Contributions

SH conducted and analyzed the experiments. MM and IK wrote the manuscript.

### Conflict of Interest Statement

The authors declare that the research was conducted in the absence of any commercial or financial relationships that could be construed as a potential conflict of interest.
